# Supplementary material for: Single-trial cross-area neural population dynamics during long-term skill learning
Source: Nat Commun. 2020 Aug 13;11:4057. doi: 10.1038/s41467-020-17902-1 (PMC7426952; doi:10.1038/s41467-020-17902-1)
Supplement: Supplementary file 1 — Supplementary Information [file 41467_2020_17902_MOESM1_ESM.pdf]

Supplementary Materials

Single-trial cross-area neural population dynamics
during long-term skill learning

Veuthey et al.

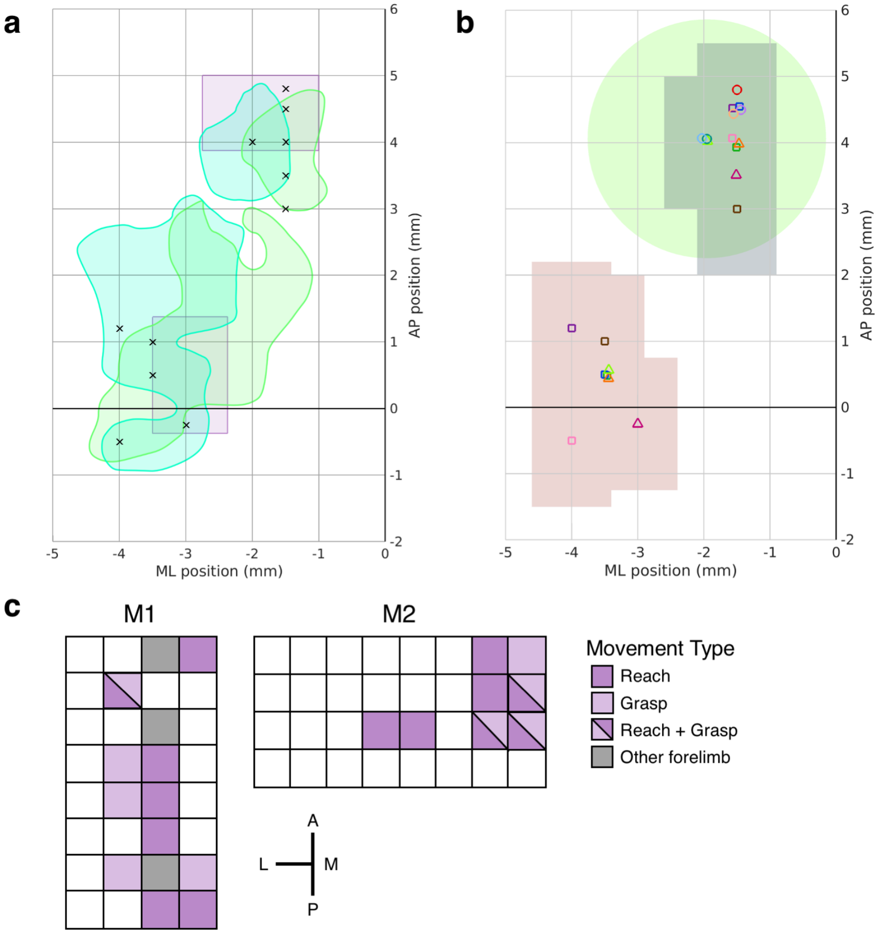

Supplementary Figure 1. M1 and M2 implantation coordinates. (a) Stimulation-based coordinates for M1 and M2 in Long-Evans rats. Shown in green and teal are regions where ICMS evoked forelimb movement in the two Long-Evans rats reported in Neafsey et al. 1986[1]; the maps were translated from diagrams of insufflated brains to flat coordinates. Shown in purple are array locations for two animals from our lab, each with a single array implanted in either M1 or M2. Electrical stimulation on these arrays evoked forelimb movements for both animals while under anesthesia. Shown as black x marks are the implantation coordinates for animals in the present study (see Supplementary Table 2). All reaching animals were implanted contralateral to their dominant hand, but coordinates for right hemisphere implants have been transposed onto the left hemisphere. Anterior medial sites are for M2 arrays, while posterior lateral sites are for M1 arrays. We observed neural responses evoked by awake, behaving forelimb movement across both arrays in all experimental animals. **(b)** M2 muscimol spread included most M2 recording sites and excluded all M1 recording sites. Colored markers indicate center of implantation sites for experimental animals. Unlike (a), markers have been jittered at sites where multiple animals had an implant. Marker color corresponds to animal ID, and marker shape indicates animal cohort (see Supplementary Table 2 for detailed legend). Green shaded circle indicates estimated M2 muscimol spread. The center of the circle is placed at the surgical coordinates for the two animals that underwent acute injection only (see Methods), and the radius of the circle is calculated based on histology from these animals (see Supplementary Figure 6). The gray shaded region shows the combined silhouette of all M2 arrays, and the red shaded region shows the combined silhouette of all M1 arrays. As in (a), all right hemisphere implants were transposed to the left hemisphere. **(c)** Grids represent the arrays shown in purple in (a), with color indicating channels where stimulation evoked reach-like, grasp-like, or other forelimb movement. Arrays are shown in the same orientation as in (a), dimensions are not to scale.

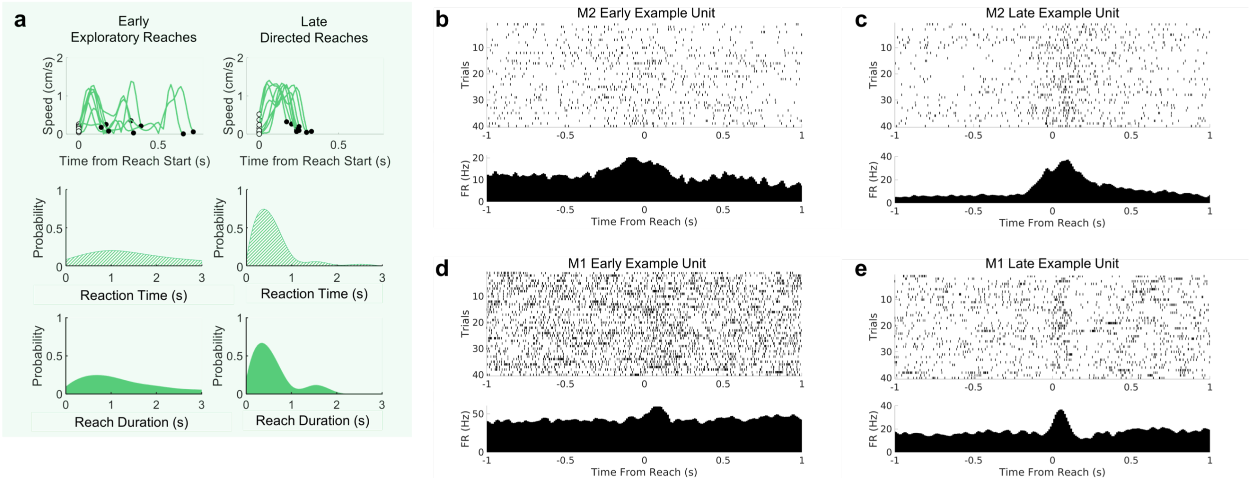

Supplementary Figure 2. Elaboration of reach-to-grasp learning behavior. (a) (Top) Speed profile for example trials in early (left) exploratory reaches and late (right) directed reaches. White dot marks reach start time. Black dot marks reach end time. Single-trial reach duration is driven by efficiency of reach targeting rather than maximal reaching speed. (Middle) Probability distribution of reaction times in early (left) exploratory reaches and late (right) directed reaches for example animal. (Bottom) Probability distribution of reach durations in early (left) exploratory reaches and late (right) directed reaches for example animal. For further details, see Supplementary Table 1. **(b)** Raster and PETH for example task-modulated unit from M2 in early learning. **(c)** Raster and PETH for example task-modulated unit from M2 in late learning. This unit was recorded on the same channel as (b). **(d)** Raster and PETH for example task-modulated unit from M1 in early learning, from same animal as (b,c). **(e)** Raster and PETH for example task-modulated unit from M1 in late learning. This unit was recorded on the same channel as (d).

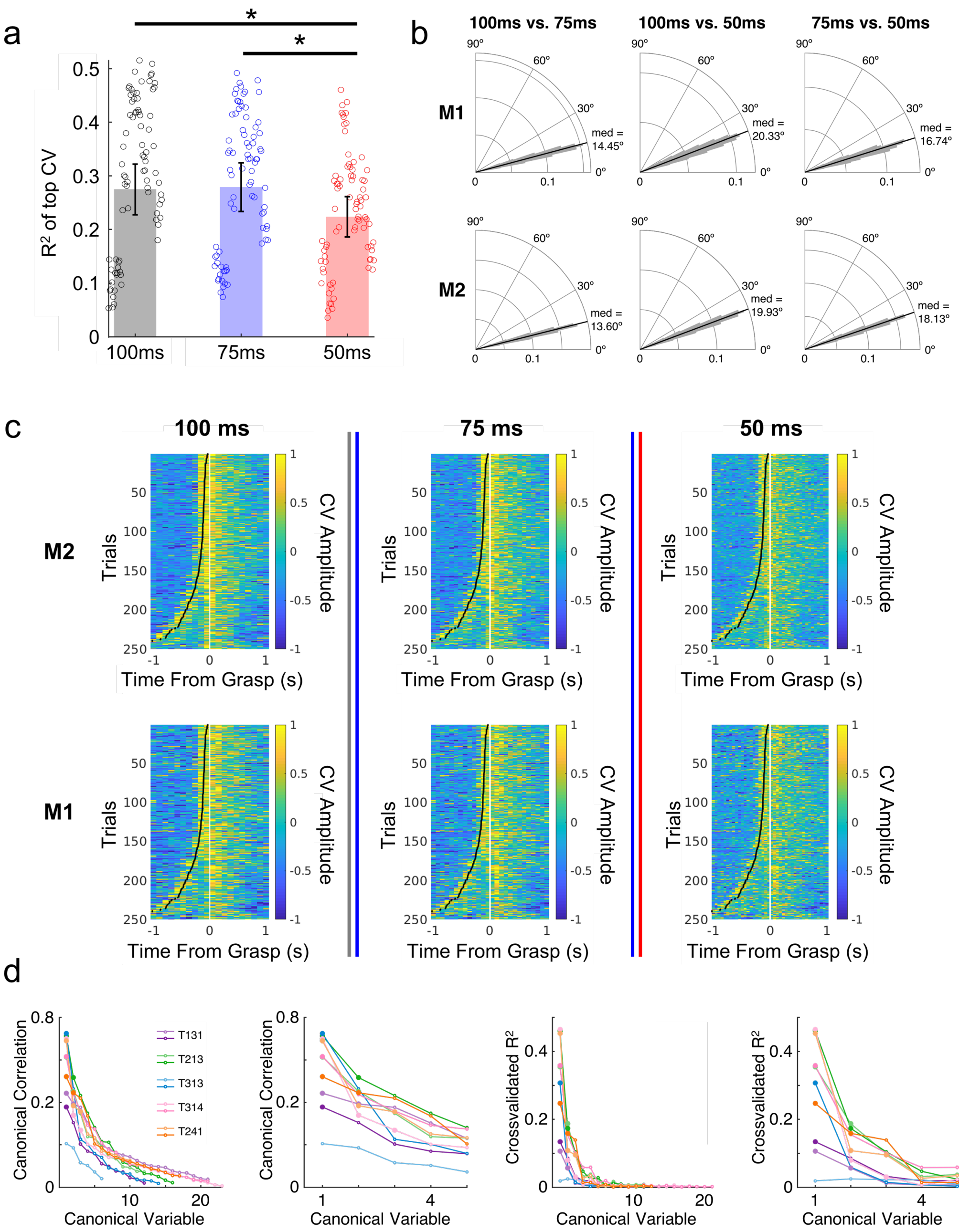

Supplementary Figure 3. Fitting CCA with different bin-widths. (a) CCA models were fit to data binned at 100, 75, and 50ms. For each dataset and model, R^2 values of the top CV were computed on 10 folds of data (90% of time bins 10 ways, held out time bins were randomly chosen and non-overlapping). Bars show mean ± std. dev. R^2 values for all models fit to data at a particular binwidth, open circles show R^2 values for individual models (4 animals x 2 sessions x 10 folds = 80 models per bin width). R^2 values were not significantly different between the models fit on 100ms data and the models fit on 75ms data (p=0.83), but were significantly lower for the models fit on 50ms data (p=0.016 for 100ms vs 50ms; p<0.0002 for 75ms vs 50ms). *, p<0.05; ***, p<0.001; two-sided hierarchical bootstraps, not adjusted for multiple comparisons. **(b)** Angle between the top CVs of CCA models fit on 100ms, 75ms, or 50ms binned data. Shaded circular histogram shows bootstrap distribution of angles, black line shows median value of bootstrap distribution. Radius indicates proportion of bootstrap samples belonging to a bin. For all comparisons, the angle between models was much smaller than 90º, indicating that models fit on different

binwidths indentified similar patterns of covariation. For M2 (bottom), the angle between the 100ms and 75ms models (left) was significantly smaller than the angle between the 100ms and 50ms models (center) or between the 75ms and 50ms models (right). For M1 (top), the angle between the 100ms and 75ms models was significantly smaller than the angle between the 100ms and 50ms models, but not significantly smaller than the angle between the 75ms and 50ms models. **(c)** Single trial trajectories were qualitatively similar at different bin widths. Data shown is the same time range the models were fit on. Trials are sorted by duration and max-normalized for visualization. **(d)** Canonical correlation values (left two plots) and cross-validated R^2 values (right two plots) across canonical variables. For both metrics, values drop off at a low dimensionality. Larger, filled circles indicate CVs that were statistically significant relative to models fit on trial-shuffled data (see Methods). Light colors indicate early learning datasets, dark colors indicate late learning datasets.

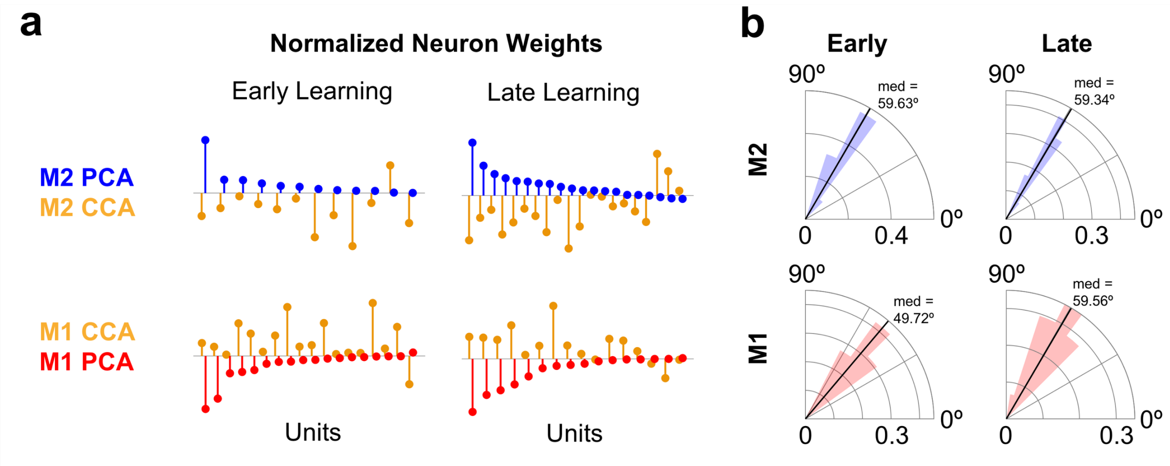

Supplementary Figure 4. PCA identifies a mix of local and cross-area dynamics. (a) Length of stems indicate weights for each neuron's contribution to local or cross-area activity, derived using PCA and CCA respectively. Neuron weights were normalized by the maximum value for any neuron in that subspace. PCA and CCA weights are shown offset and opposing for visual clarity; for all models, more neurons had positive weights than negative. M2 and M1 subspace neuron weights in early (left) learning and late (right) learning. **(b)** Angle in multi-dimensional neural space between PCA and CCA subspaces for M2 (top) and M1 (botom), for both early (left) and late (right) learning. Shaded histograms show bootstrap distribution of angles, solid black lines show median value of the bootstrap distribution. Radius indicates the proportion of bootstrap values in each bin.

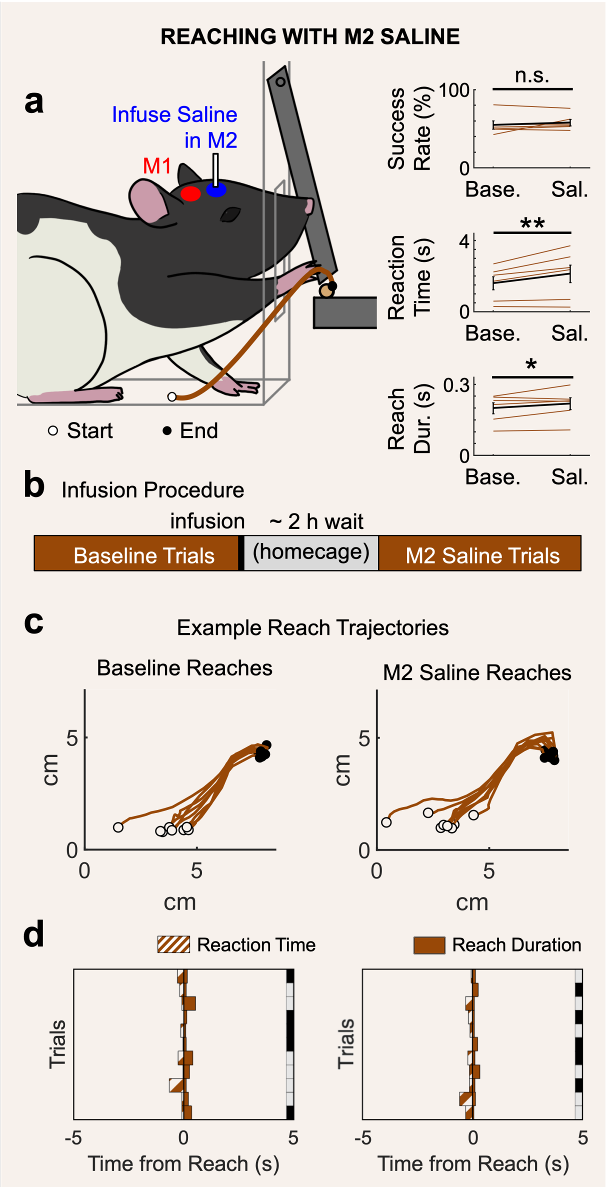

Supplementary Figure 5. M2 saline infusions do not severely affect learned reach behavior. (a) (Left) Rats previously trained on the reach-to-grasp task were infused with saline in M2. (Right) M2 saline increased reaction time (p=0.0021) and reach duration (p=0.0452), but did not decrease success rate (p=0.7453). Brown lines show values for individual animals, black lines show mean ± std. dev. *, p<0.05; **, p<0.01, hierarchical bootstraps, one-sided, not adjusted for multiple-comparisons. **(b)** Experimental paradigm for evaluation of reach behavior during M2 saline infusion. **(c)** Example reach from a single animal during baseline (left) and M2 saline infusion (right). **(d)** Example consecutive single-trial representations of reaction time and reach duration. Right border of plot shows accuracy, with success in grey and failure in black.

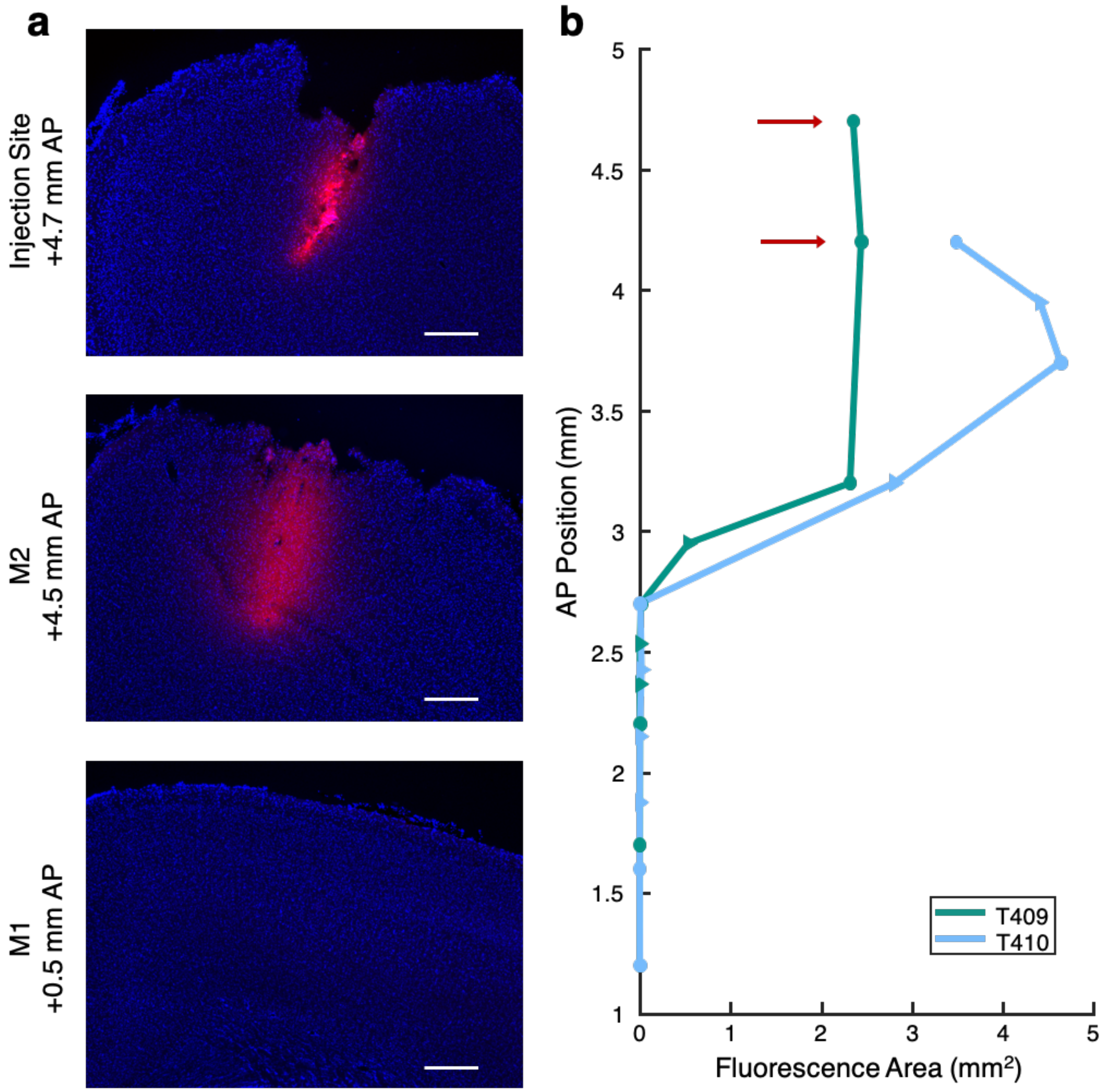

Supplementary Figure 6. Histological verification of M2 muscimol spread. (a) Images showing fluorescent muscimol spread at the injection site (top), M2 (center), and M1 (bottom) in one acute injection animal (T409, see Methods). AP position is reported by comparison to brain atlas[2]. Fluorescent muscimol is shown in red, DAPI in blue. Scale bar represents 500 µm. **(b)** Estimated muscimol spread was measured independently in two animals, with similar results. Slices were imaged starting with the injection site (determined based on tissue damage) and moving posteriorly until no fluorescence was seen for several slices. Fluorescence area was calculated using ImageJ after correcting to a baseline fluorescence value from a distant slice. Circles indicate slices where AP position was determined with reference to a brain atlas. Triangles indicate slices where AP position was inferred based on slice width and atlas-referenced slices. In both animals, fluorescence was close to zero starting at +2.7 AP. By averaging the two animals, we estimated muscimol spread to be 1.8mm from the injection site. Red arrows indicate the slices shown in (a).

Rat ID	Condition	Day	Camera Framerate	Trials Offered	Completed Trials	Successful Trials	Success Rate	Reaction Time	Reach Duration
**Learning Cohort** ***(Reach training + neural recordings, no inactivation)***									
T131	Early	Training day 1	30 Hz	313	183	43	23.50%	4.39 (0.15 - 14.73) s	0.23 (0.03 - 1.07) s
	Late	Training day 3		315	291	144	49.49%	0.27 (0.15 - 11.84) s	0.21 (0.10 - 0.77) s
T213	Early	Training day 1	75 Hz	224	211	71	33.65%	0.34 (0.00 - 14.92) s	0.11 (0.00 - 0.56) s
	Late	Training day 3		301	298	178	59.73%	0.20 (0.00 - 7.52) s	0.08 (0.00 - 0.32) s
T241	Early	Training day 1	75 Hz	250	184	50	27.17%	3.02 (0.32 - 24.18) s	0.34 (0.07 - 2.00) s
	Late	Training day 3		300	273	162	59.34%	0.58 (0.03 - 15.76) s	0.32 (0.06 - 0.91) s
T313†	Early	Training day 2	100 Hz	87	38	15	39.47%	11.73 (2.51 - 29.69) s	0.18 (0.06 - 0.66) s
	Late	Training day 15		213	211	133	63.03%	0.33 (0.00 - 13.34) s	0.15 (0.05 - 0.81) s
T314	Early	Training day 1	100 Hz	299	142	28	19.72%	5.96 (0.41 - 24.39) s	0.38 (0.11 - 1.22) s
	Late	Training day 10		259	250	145	58.00%	0.42 (0.00 - 12.62) s	0.11 (0.02 - 0.62) s
**Inactivation-Only Cohort** ***(Reach training + infusion cannulas, no neural recordings)***									
T282	Baseline (M)	Re-training day 7	75 Hz	100	97	70	72.17%	0.37 (0.00 - 4.03) s	0.17 (0.01 - 0.40) s
	M2 Muscimol			99	94	42	44.68%	0.59 (0.00 - 5.44) s	0.19 (0.01 - 0.85) s
	Baseline (S)	Re-training day 7		100	93	75	80.65%	0.04 (0.00 - 9.76) s	0.23 (0.01 - 0.49) s
	M2 Saline			100	97	74	76.29%	0.09 (0.00 - 16.23) s	0.23 (0.04 - 0.80) s
T291	Baseline (M)	Re-training day 8	100 Hz	106	105	45	42.86%	0.16 (0.00 - 6.48) s	0.11 (0.01 - 0.34) s
	M2 Muscimol			105	90	31	34.44%	0.77 (0.00 - 11.15) s	0.16 (0.02 - 0.59) s
	Baseline (S)	Re-training day 6		100	100	50	50.00%	0.24 (0.00 - 2.31) s	0.10 (0.02 - 0.48) s
	M2 Saline			100	100	53	53.00%	0.20 (0.00 - 6.32) s	0.10 (0.03 - 0.34) s
T315	Baseline (M)	Re-training day 2	75 Hz	100	98	65	66.33%	0.94 (0.55 - 8.65) s	0.21 (0.03 - 0.51) s
	M2 Muscimol			100	82	34	41.46%	2.35 (0.89 - 17.05) s	0.37 (0.03 - 0.91) s
	Baseline (S)	Re-training day 2		111	101	50	49.51%	1.79 (0.88 - 13.81) s	0.25 (0.04 - 0.72) s
	M2 Saline			139	125	60	48.00%	2.13 (0.84 - 16.47) s	0.29 (0.03 - 0.87) s
**Inactivation + Recording Cohort** ***(Reach training + infusion cannulas + neural recordings)***									
T336	Baseline (M)	Re-training day 2	63.58 Hz	99	91	38	41.76%	1.47 (0.63 - 11.76) s	0.22 (0.06 - 0.51) s
	M2 Muscimol			100	93	20	21.51%	4.82 (0.94 - 13.40) s	0.30 (0.09 - 1.00) s
	Baseline (S)	Re-training day 2		106	79	44	55.70%	1.36 (0.66 - 6.94) s	0.23 (0.03 - 0.39) s
	M2 Saline			149	118	66	55.93%	1.93 (0.83 - 12.52) s	0.22 (0.05 - 0.44) s
T349	Baseline (M)	Re-training day 3	63.58 Hz	97	97	61	62.89%	1.07 (0.55 - 7.05) s	0.19 (0.06 - 0.58) s
	M2 Muscimol			199	81	13	16.05%	5.00 (0.31 - 13.89) s	0.36 (0.15 - 0.72) s

	Baseline (S)	Re-training day 3		109	103	44	42.72%	1.44 (0.00 - 13.86) s	0.23 (0.06 - 0.52) s
	M2 Saline			99	96	60	62.50%	1.61 (0.64 - 12.79) s	0.23 (0.06 - 0.42) s
T391	Baseline (M)	Re-training day 3	100 Hz	113	89	48	53.93%	0.91 (0.00 - 7.56) s	0.15 (0.028 - 0.55) s
	M2 Muscimol			136	85	56	65.88%	1.57 (0.00 - 15.27) s	0.17 (0.04 - 2.74) s
	Baseline (S)	Re-training day 3		112	92	48	52.17%	1.91 (0.00 - 12.33) s	0.13 (0.05 - 0.66) s
	M2 Saline			122	94	51	54.26%	2.50 (0.04 - 16.99) s	0.13 (0.05 - 1.66) s

Supplementary Table 1. Behavior summary. For the learning cohort, training day is measured from the first day the rat is exposed to the reaching task (excluding small number of handedness trials performed prior to implantation, see Methods). For the inactivation cohorts, animals received extensive reach training (often > 14 days) prior to their implantation and use in this study, however several of the animals also experienced extended periods of no reach training, and were therefore retrained to plateau performance prior to the muscimol and saline experiments. For these animals, training day is measured from the beginning of the most recent bout of training (same training bout for muscimol and saline experiments). For all animals, reaction time and reach duration are reported as *median (min – max)*, and measured in seconds. [†]Rat T313 is the animal excluded from most of the neural analyses due to having no significant CVs in the Early condition (see Methods).

Rat ID	Handedness	Implant	Stereotaxic coordinates (mm)	Marker in Supp. Fig. 1b
**Learning Cohort** *(Reach training + neural recordings, no inactivation)*				
T131	Left	M2 array	ML: 1.5; AP: +4.5; DV: -1.8	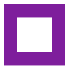
		M1 array	ML: 4.0; AP: +1.2; DV: -1.7	
T213	Left	M2 array	ML: 1.5; AP: +4.0; DV: -1.5	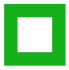
		M1 array	ML: 3.5; AP: +0.5; DV: -1.5	
T241	Right	M2 array	ML: 1.5; AP: +4.5; DV: -1.5	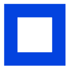
		M1 array	ML: 3.5; AP: +0.5; DV: -1.5	
T313†	Right	M2 array	ML: 1.5; AP: +4.0; DV: -1.5	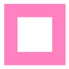
		M1 array	ML: 4.0; AP: -0.5; DV: -1.5	
T314	Right	M2 array	ML: 1.5; AP: +3.0; DV: -1.5	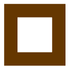
		M1 array	ML: 3.5; AP: +1.0; DV: -1.4	
**Inactivation-Only Cohort** *(Reach training + infusion cannulas, no neural recordings)*				
T282	Right	M2 cannula L	ML: 1.5; AP: +4.8; DV: -1.7	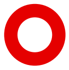
		M2 cannula R	ML: 1.5; AP: +4.5; DV: -1.5	
T291	Right	M2 cannula L	ML: 1.5; AP: +4.5; DV: -1.7	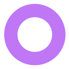
		M2 cannula R	ML: 1.5; AP: +4.5; DV: -1.5	
T315	Right	M2 cannula L	ML: 1.5; AP: +4.5; DV: -1.7	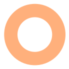
		M2 cannula R	ML: 1.5; AP: +4.5; DV: -1.5	
**Inactivation + Recording Cohort** *(Reach training + infusion cannulas + neural recordings)*				
T336	Right	M2 array	ML: 1.5; AP: +4.0; DV: -1.6	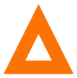
		M2 cannula	ML: 2.5; AP: +4.0; DV: -1.6	
		M1 array	ML: 3.5; AP: +0.5; DV: -1.6	
T349	Right	M2 array	ML: 1.5; AP: +3.5; DV: -1.6	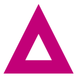
		M2 cannula	ML: 2.0; AP: +3.5; DV: -1.6	
		M1 array	ML: 3.0; AP: -0.25; DV: -1.6	
T391	Right	M2 array	ML: 2.0; AP: +4.0; DV: -1.6	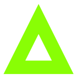
		M2 cannula	ML: 2.5; AP: +4.0; DV: -1.6	
		M1 array	ML: 3.5; AP: +0.5; DV: -1.6	
**Acute Fluorescent Muscimol Injection Cohort**				
T409	N/A	M2 injection L	ML: 2.0; AP: +4.0; DV: -1.5	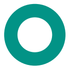
		M2 injection R	ML: 2.0; AP: +4.0; DV: -1.5	

T410	N/A	M2 injection L	ML: 2.0; AP: +4.0; DV: -1.5	
		M2 injection R	ML: 2.0; AP: +4.0; DV: -1.5	

Supplementary Table 2. Implantation coordinates for all experimental animals.

SUPPLEMENTARY REFERENCES

1. Neafsey, E. J. *et al.* The organization of the rat motor cortex: A microstimulation mapping study. *Brain Research Reviews* **11**, 77–96 (1986).

2. Paxinos, G., & Watson, C. (1986). The rat brain in stereotaxic coordinates, 2nd edR Academic Press. New York.
