# Supplementary material for: Single-trial cross-area neural population dynamics during long-term skill learning
Source: Nat Commun. 2020 Aug 13;11:4057. doi: 10.1038/s41467-020-17902-1 (PMC7426952; doi:10.1038/s41467-020-17902-1)
Supplement: Supplementary file 3 — Reporting Summary [file 41467_2020_17902_MOESM3_ESM.pdf]

nature research

Corresponding author(s): Karunesh GANGULY

Last updated by author(s): 2020/07/12

Reporting Summary

Nature Research wishes to improve the reproducibility of the work that we publish. This form provides structure for consistency and transparency in reporting. For further information on Nature Research policies, see Authors & Referees and the Editorial Policy Checklist .

Statistics

For all statistical analyses, confirm that the following items are present in the figure legend, table legend, main text, or Methods section.

n/a	Confirmed	
☐	☒	The exact sample size (*n*) for each experimental group/condition, given as a discrete number and unit of measurement
☐	☒	A statement on whether measurements were taken from distinct samples or whether the same sample was measured repeatedly
☐	☒	The statistical test(s) used AND whether they are one- or two-sided<br>*Only common tests should be described solely by name; describe more complex techniques in the Methods section.*
☐	☒	A description of all covariates tested
☐	☒	A description of any assumptions or corrections, such as tests of normality and adjustment for multiple comparisons
☐	☒	A full description of the statistical parameters including central tendency (e.g. means) or other basic estimates (e.g. regression coefficient) AND variation (e.g. standard deviation) or associated estimates of uncertainty (e.g. confidence intervals)
☐	☒	For null hypothesis testing, the test statistic (e.g. *F*, *t*, *r*) with confidence intervals, effect sizes, degrees of freedom and *P* value noted<br>*Give P values as exact values whenever suitable.*
☒	☐	For Bayesian analysis, information on the choice of priors and Markov chain Monte Carlo settings
☐	☒	For hierarchical and complex designs, identification of the appropriate level for tests and full reporting of outcomes
☒	☐	Estimates of effect sizes (e.g. Cohen's *d*, Pearson's *r*), indicating how they were calculated

Our web collection on statistics for biologists contains articles on many of the points above.

Software and code

Policy information about availability of computer code

Data collection	We use MATLAB 2014a and the Adafruit Motor Library (V1) to run the automated rodent reaching box (Wong et al., 2015). We use TDT OpenEx Software Suite v2.30 and v2.31 to collect neurophysiology data.
Data analysis	We used MATLAB 2015b, 2018b, and 2019b for data analyses in this manuscript. Custom scripts were developed for analyses specific to the animals/behavioral paradigms analyzed here, and will be made available on request. We also used DataHigh v1.2 for Factor Analysis. We used WaveClus 2.0 and MountainSort v2 for spike sorting. We used ImageJ v1.47 for image analysis.

For manuscripts utilizing custom algorithms or software that are central to the research but not yet described in published literature, software must be made available to editors/reviewers. We strongly encourage code deposition in a community repository (e.g. GitHub). See the Nature Research guidelines for submitting code & software for further information.

Data

Policy information about availability of data

All manuscripts must include a data availability statement. This statement should provide the following information, where applicable:

- Accession codes, unique identifiers, or web links for publicly available datasets
- A list of figures that have associated raw data
- A description of any restrictions on data availability

The list of figures and associated data are available in the included supplementary source data file.

Field-specific reporting

Please select the one below that is the best fit for your research. If you are not sure, read the appropriate sections before making your selection.

Life sciences study design

All studies must disclose on these points even when the disclosure is negative.

Sample size	Behavior sample sizes were chosen based on previous experiments we conducted in stroke animals (Gulati et al., J. Neurosci, 2014). In that paper, the behavioral changes observed after stroke were significantly large enough that 4 animals/group were sufficient to perform the relevant analyses. Thus, for the learning cohort we ensured there were at least 4 animals/group (for some experiments, we conducted experiments in greater than 4 animals).<br><br>Muscimol inactivation behavioral data was collected from 6 animals, with additional neural recording in 3 animals.
Data exclusions	In the learning cohort, one animal was excluded from all CCA analyses because the number of recorded neurons was insufficient to perform the analyses.
Replication	We have presented all of the data we collected in the paper. All learning and lesion effects were investigated in multiple animals to ensure replicability. Our results are consistent with previous work observing behavioral changes with learning and lesions (e.g., behavioral improvement with reach-to-grasp learning, behavioral impairment with M2 muscimol infusions). Given this, there were no other attempts to reproduce the data as it would have been an additional use of animals and other resources, and we would not expect additional data to change our fundamental conclusions.
Randomization	- Randomization is the most relevant to the muscimol inactivation experiments. However, because of the within-day task design and concerns about muscimol washout time, we could not randomize the order of the baseline and muscimol blocks. To address this, we performed saline control experiments.<br>- Animals were sequentially allocated into learning vs M2 inactivation vs imaging experimental groups. As we collected data with automated behavioral boxes, this work did not require the random allocation of experimental subjects.
Blinding	The behavioral task is fully automatic once started and does not require experimenter intervention. The metric of accuracy (whether the animal retrieved the pellet or not) is also quite objective.<br><br>For muscimol inactivation experiments, because of the within-day task design, experimenters were unable to be blinded to task condition. To address this, we performed saline control experiments.

Reporting for specific materials, systems and methods

We require information from authors about some types of materials, experimental systems and methods used in many studies. Here, indicate whether each material, system or method listed is relevant to your study. If you are not sure if a list item applies to your research, read the appropriate section before selecting a response.

Materials & experimental systems

n/a	Involved in the study
☒	☐ Antibodies
☒	☐ Eukaryotic cell lines
☒	☐ Palaeontology
☐	☒ Animals and other organisms
☒	☐ Human research participants
☒	☐ Clinical data

Methods

n/a	Involved in the study
☒	☐ ChIP-seq
☒	☐ Flow cytometry
☒	☐ MRI-based neuroimaging

Animals and other organisms

Policy information about studies involving animals; ARRIVE guidelines recommended for reporting animal research

Laboratory animals	In this experiment, we used male Long-Evans rats. There were housed in a 12h:12h light/dark cycle room. Animals were purchased as 3-4 months old adults between 250 - 400 grams.
Wild animals	No wild animals were involved in this study
Field-collected samples	This study did not involve samples collected from the field
Ethics oversight	All procedures were in accordance with protocols approved by the Institutional Animal Care and Use Committee at the San Francisco Veterans Affairs Medical Center

Note that full information on the approval of the study protocol must also be provided in the manuscript.
